# Solitary Renal Metastasis Arising from a Pulmonary Adenoid Cystic Carcinoma: A Case Report and Review of the Literature

**DOI:** 10.1155/2021/8863475

**Published:** 2021-03-30

**Authors:** Prodromos Philippou, Anastasios Michalakis, Maria Miliatou, Christiana Poullou, Pavlos Constantinou

**Affiliations:** ^1^St George's Medical School, University of Nicosia, Nicosia, Cyprus; ^2^Apollonion Hospital, Nicosia, Cyprus; ^3^251 Airforce Hospital, Athens, Greece; ^4^Histopathology and Cytology Laboratory Services, Nicosia, Cyprus; ^5^School of Medicine, European University of Cyprus, Cyprus

## Abstract

Adenoid cystic carcinoma (ACC) is a malignancy affecting the salivary glands and rarely involving the lung. Due to its rarity, primary lung ACC remains incompletely understood. We herein report the case of a 57-year-old female patient who was initially diagnosed with primary lung ACC and was treated by lobectomy. Seven years later, an abdominal computed tomography scan performed in the context of surveillance revealed the presence of a solid lesion arising from the lower pole of the left kidney. The patient underwent left partial nephrectomy, and histopathology confirmed a completely excised metastatic ACC.

## 1. Introduction

Adenoid cystic carcinoma (ACC) is a rare form of malignancy that arises within secretory glands, most commonly the major and minor salivary glands of the head and neck. Other sites of origin include lacrimal glands, breast, skin, and vulva [1]. ACC tends to metastasize to the lung and, less frequently, to the brain, liver, and bones [2]. Primary salivary-type tumors including ACC account for approximately 0.1% to 0.2% of all lung cancers and are histologically indistinguishable from their salivary gland counterparts [2]. They originate from the submucosal glands of the tracheobronchial tree and usually behave as low-grade malignancies with better prognosis compared to conventional lung cancer. Due to its rarity, primary lung ACC remains incompletely understood, and its clinical behavior, optimal treatment, and long-term outcome have yet to be clarified.

Renal metastases have been described in other malignancies such as lung, breast, and gastric carcinoma. Metastatic involvement of the kidney is usually a feature of disseminated disease, while solitary renal metastases are less frequent and mimic primary renal tumors [3]. In patients harboring another malignancy, solitary renal masses may represent synchronous primary tumors of the kidney.

Herein, we report the case of a 57-year-old female patient who presented with a solitary renal metastasis of a lung ACC and was successfully managed by minimally invasive nephron-sparing surgery. We report this case to highlight its uniqueness in terms of primary site (lung) and natural history (metachronous solitary metastasis to the kidney), and we focus on relevant issues such as differential diagnosis, the role of organ-sparing metastectomy, and the need for long-term follow-up of this rare malignancy.

## 2. Case Presentation

**Figure 1:**
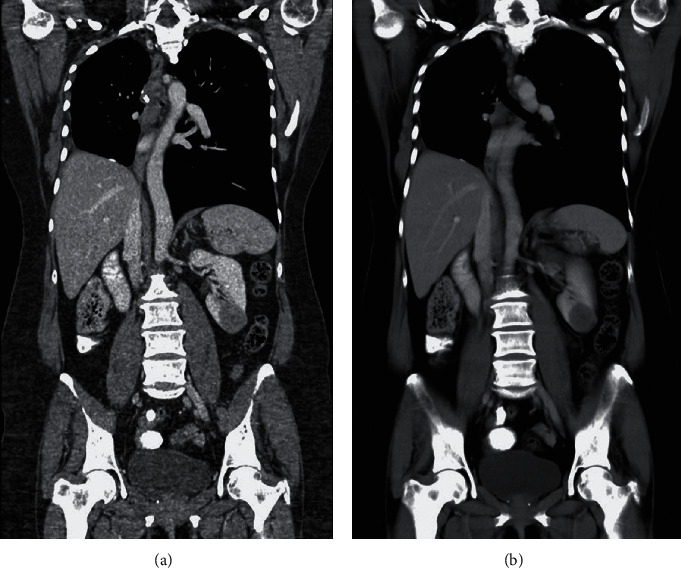
Contrast-enhanced computed tomography in arterial (a) and venous (b) phases, revealing a slightly enhancing mass occupying the lower pole of the left kidney.

**Figure 2:**
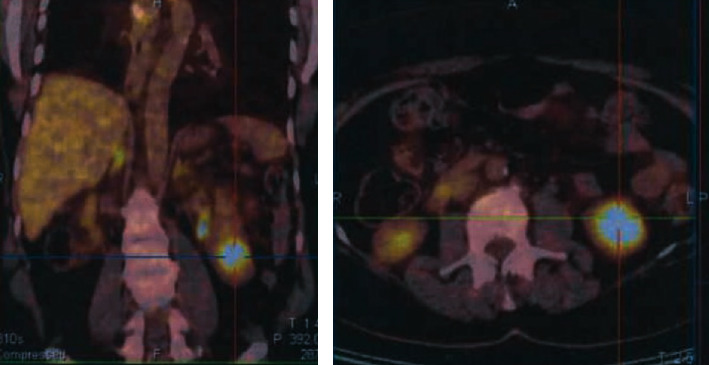
^18^F-Fluorodeoxyglucose positron emission tomography-computed tomography fusion scan images show a well-defined, heterogeneously low-grade fluorodeoxyglucose avid solid hypodense lesion at the lower pole of the left kidney.

A 57-year-old asymptomatic female patient was referred for a 33 × 27 mm solid lesion arising from the lower pole of the left kidney, which was diagnosed by computed tomography (CT) performed in the context of annual surveillance (Figure 1). She had been diagnosed with primary lung ACC seven years earlier and underwent right lower lobectomy, without any further adjuvant treatment. On precontrast CT images, the lesion appeared well-circumscribed, homogeneous, and relatively hypodense (30 +/-5 HU). It enhanced slightly in the arterial phase (45 +/-5 HU), but the enhancement was more pronounced in the venous phase (65 +/-5 HU). On 18F-fluorodeoxyglucose positron emission tomography–CT scan (Figure 2), a lump-shaped radioactive concentration shadow was observed at the lower pole of the left kidney, with a maximum standardized uptake value of 7. No other metastatic lesions were detected. Based on imaging characteristics and the patient's history, the differential diagnosis included nonclear cell renal cell carcinoma, renal metastasis, and benign renal tumors.

According to the Oncology Department's protocol for small renal masses (≤4 cm in greatest dimension) of questionable origin, percutaneous CT-guided biopsy of the renal lesion was performed, and histopathological analysis revealed a pattern compatible with ACC, similar to the primary lung tumor. Given the patient's age and the features of the tumor, nephron-sparing surgery was recommended. The patient underwent laparoscopic transperitoneal partial nephrectomy. The postoperative course was uneventful, and the patient was discharged on postoperative day two.

**Figure 3:**
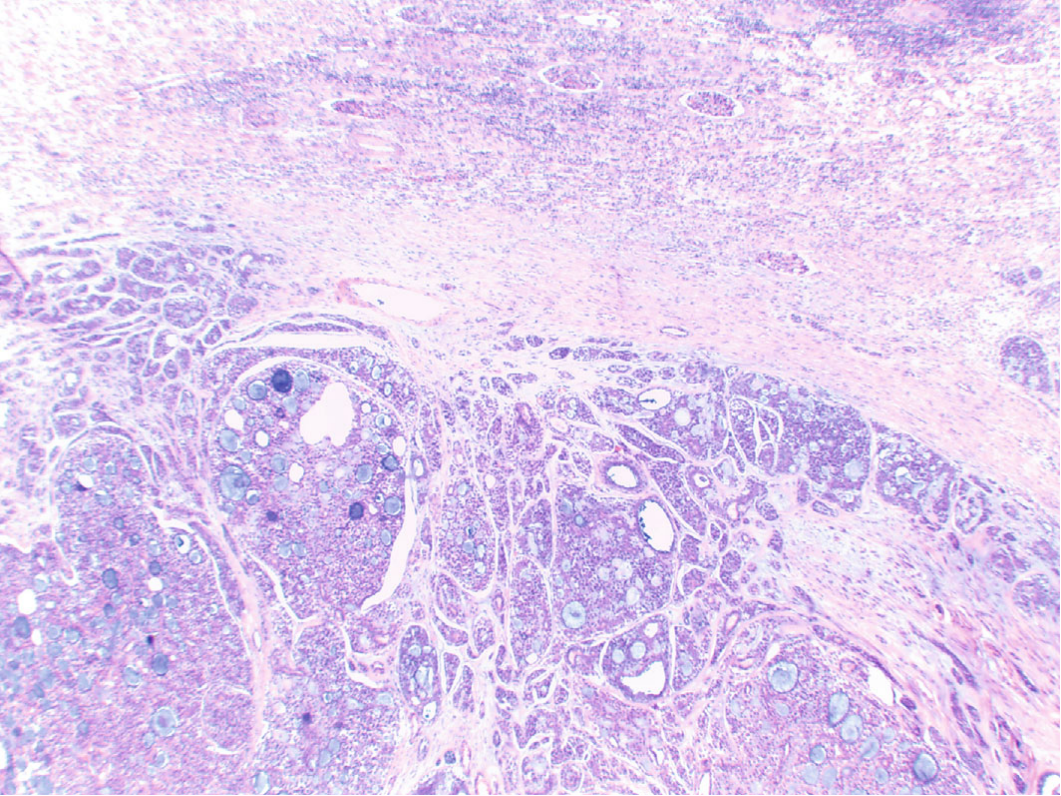
Metastatic adenoid cystic carcinoma to the kidney, showing the characteristic cribriform histological pattern of growth. Normal renal parenchyma is seen at the upper half of the image (haematoxylin-eosin stain, original magnification 40x).

Gross examination of the specimen revealed a well-circumscribed, solid, and whitish tumor, measuring 37 × 39 × 35 mm. Microscopically, tumor cells were arranged in typical cribriform, tubular, and focally solid growth patterns. Neoplastic cells were small with scant cytoplasm. The stroma manifested myxoid and hyalinized features surrounded by tumor cells forming cylindrical structures (Figure 3). Immunohistochemically, neoplastic cells showed focal cytoplasmic or very weak nuclear expression of PAX8 which was considered a negative result. The strong nuclear staining of the renal tubular epithelium was used as an internal positive control. PAX8 negativity confirmed the extrarenal and extra-Müllerian origin of the tumor. The epithelial cells showed positivity for CD117 and CK7, and the myoepithelial cells showed positivity for P63, highlighting the dual cell population of the neoplasm, a typical feature of adenoid cystic carcinoma. Surgical margins were negative. These findings confirmed a completely excised metastatic ACC. Follow-up imaging at 24 months showed no evidence of recurrent disease.

## 3. Discussion

Historically, metastatic involvement of the kidney was considered a rare phenomenon. According to autopsy studies, the rate of renal metastases ranges from 2.4% to 7.2% [4], while detection of renal metastases in the clinical setting is less frequent [5, 6]. The most common sites of origin for tumors that metastasize to the kidney are the lung, gastrointestinal tract, breast, ovary, soft tissue, and thyroid [5]. Most patients diagnosed with renal metastases are either asymptomatic or present with nonspecific findings, such as albuminuria or microscopic hematuria [7]. In the case presented herein, the patient was diagnosed in the context of surveillance and was completely asymptomatic.

Metastatic disease involving the kidney usually presents with bilateral and multiple lesions, associated with widespread dissemination of the primary tumor [8]. Solitary renal metastases, however, are uncommon [5], and most of these patients are diagnosed by routine imaging. Differentiation between a solitary renal metastasis and a primary renal tumor based on imaging can be challenging. Characteristics on CT scan suggestive of renal metastasis include endophytic growth, either isodensity or slightly lower attenuation compared to the normal renal parenchyma, hypovascularity ,and slight enhancement on contrast-enhanced images [3]. The imaging findings in this case are in accordance with these features.

In the past, the presence of a renal lesion in a patient with a history of extrarenal malignancy was considered an absolute indication for biopsy due to major treatment implications [5–9]. This approach, however, has been questioned in the context of solitary renal masses [5]. According to a previous retrospective study [8], decision-making in this setting can be based on the status of the extrarenal malignancy and the imaging characteristics of the renal lesion. The two major predictive factors for renal metastasis are evidence of clinical progression and/or other metastases from the nonrenal malignancy and lack of enhancement of the renal mass on imaging. The absence of progression of the nonrenal primary is considered a predictive factor for a renal primary over a solitary renal metastasis. In the case presented herein, the imaging characteristics of the lesion (endophytic, slightly low in attenuation, slight enhancement after intravenous contrast administration) were more typical of a metastatic lesion. The primary lesion, however, had been in remission, and no other metastatic focus was identified on imaging. The decision to perform percutaneous biopsy prior to surgical extirpation of the renal lesion in this case was based on the absence of progression of the primary tumor, even though the imaging findings were consistent with metastasis.

ACC is characterized by an indolent clinical course, a variable distant recurrence rate (4.1-44.7%) and a tendency for late-onset metastases [10]. The organs most frequently involved by metastatic ACC are the lungs, bones, brain, and liver [2]. On reviewing the limited cases of metastatic ACC with renal involvement, the most common primary sites are major or minor salivary glands (parotid gland, submandibular gland, palate, floor of mouth, sinonasal region), lacrimal gland, and breast [11–15]. The time interval between primary diagnosis and development of renal metastasis ranged from 3 to 14 years, with an average interval of 9.8 years, representing an indolent growth pattern and a potential for late metastases [15]. To our knowledge, only four cases of pulmonary ACC metastatic to the kidney have been described in the literature to date (Table 1) [16–19]. Two of these reports describe renal involvement in the context of widespread metastatic disease with a poor outcome [17, 18]. Surgery (with or without adjuvant radiotherapy) was the most common primary treatment modality, and the time interval between initial diagnosis of lung ACC and development of renal metastasis ranged between 3 and 23 years. All previous patients presented with symptoms indicating a renal mass (i.e., hematuria and/or flank pain), but our patient was asymptomatic and was diagnosed in the context of annual surveillance.

The prognosis of patients with distant metastases following primary lung ACC varies, but previous reports have suggested that metastases have a tendency for slow growth similar to the primary tumor [2]. In this context, complete surgical excision of metastases, if feasible, should be considered as a therapeutic option, in order to achieve control of disease and prolong survival. Given the rarity of the case presented herein, however, clear recommendations cannot be made. For metastatic ACC of the salivary glands, survival is related to the disease-free interval [20], as metastectomy for metachronous metastases (interval > 36 months) appears to achieve better disease control. While surgical excision (radical or partial nephrectomy) is considered the standard of care in patients with primary renal malignancies, there are no clear guidelines for the surgical management of renal metastases. According to the limited available data [5, 6], surgical excision of renal metastases in carefully selected patients with oligometastatic disease and good performance status achieves acceptable long-term survival rates. The role of nephron-sparing surgery in these cases is not established, since the available literature only includes case reports and nonrandomized case series. According to a large single-centre retrospective series of 151 patients, only one-third of patients were candidates for surgical excision of renal secondaries, and less than 20% of them underwent organ-sparing surgery (i.e., partial nephrectomy) [5].

Local and distant metastases of ACC are frequent due to the tumor's tendency to invade the peri-neural space [2]. Currently, there is no consensus for the treatment of metastatic ACC, as cytotoxic chemotherapy produces very low response rates (5-15%) [21, 22]. Several advances in the understanding of the pathogenesis and molecular phenotype of this malignancy have been made, and new compounds (i.e., antiangiogenic agents, tailored agents, and checkpoint inhibitors) are under investigation. Genetic analyses have shown that MYB-NFIB fusion transcripts and/or MYB overexpression are observed in the majority of ACC cases [21]. In this context, the tyrosine kinase inhibitors axitinib, dovitinib, and lenvatinib exhibited clinical activity against ACC in phase II studies [23–25]. There remains a need for further studies that incorporate molecular analysis in the management of incurable ACC, as well as the development of effective novel therapeutic approaches.

## 4. Conclusion

The uniqueness of the case presented herein involves metachronous solitary metastatic renal metastasis from ACC arising at an uncommon primary site, managed by organ-sparing surgery. The patient underwent laparoscopic partial nephrectomy with negative surgical margins and remained disease-free 24 months after surgery. Since late distant metastases of ACC are not uncommon, long-term follow-up is recommended.

## Figures and Tables

**Table 1:** Summary of primary lung ACC cases with renal metastases.

Ref no	Demographics	Treatment of primary tumor	Time interval to renal metastasis (years)	Symptoms at presentation	Metastatic tumor location/size (cm)	Management of renal tumor	Oncological outcome
[16]	47 yo male	Right pneumonectomy	23 years	Hematuria	Left kidney/9.0 cm	Radical nephrectomy	No recurrence after 12 months
[17]	79 yo male	Not done	3 years	Hematuria and right flank pain	Right kidney/4.4 cm + multiple left renal lesions	Chemotherapy	Patient died 30 months after diagnosis
[18]	50 yo male	Right lower lobectomy	7 years	Flank pain	Right kidney/9.3 cm	Radical nephrectomy (cytoreductive)	Not reported
[19]	70 yo male	Right upper lobectomy + radiotherapy	3 years	Cough, fever, weight loss	Lower and upper pole of right kidney/1.8 cm	Radical nephrectomy	No recurrence after 24 months
This case	57 yo female	Right lower lobectomy	7 years	Asymptomatic	Lower pole of left kidney/3.3 cm	Partial nephrectomy	No recurrence after 24 months

## Data Availability

Previously reported data were used to support this study. These prior studies (and datasets) are cited at relevant places within the text as references.

## References

[1] Resio B. J., Chiu A. S., Hoag J., Dhanasopon A. P., Blasberg J. D., Boffa D. J. (2018). Primary salivary type lung cancers in the National Cancer Database. *The Annals of Thoracic Surgery*.

[2] Molina J. R., Aubry M. C., Lewis J. E. (2007). Primary salivary gland-type lung cancer: spectrum of clinical presentation, histopathologic and prognostic factors. *Cancer*.

[3] Bailey J. E., Roubidoux M. A., Dunnick N. R. (1998). Secondary renal neoplasms. *Abdominal Imaging*.

[4] Bracken R. B., Chica G., Johnson D. E., Luna M. (1979). Secondary renal neoplasms: an autopsy study. *Southern Medical Journal*.

[5] Zhou C., Urbauer D. L., Fellman B. M. (2016). Metastases to the kidney: a comprehensive analysis of 151 patients from a tertiary referral centre. *BJU International*.

[6] Adamy A., Von Bodman C., Ghoneim T., Favaretto R. L., Bernstein M., Russo P. (2011). Solitary, isolated metastatic disease to the kidney: Memorial Sloan-Kettering Cancer Center experience. *BJU International*.

[7] Choyke P. L., White E. M., Zeman R. K., Jaffe M. H., Clark L. R. (1987). Renal metastases: clinicopathologic and radiologic correlation. *Radiology*.

[8] Sánchez-Ortiz R. F., Madsen L. T., Bermejo C. E. (2004). A renal mass in the setting of a nonrenal malignancy: when is a renal tumor biopsy appropriate?. *Cancer*.

[9] Sahni V. A., Silverman S. G. (2009). Biopsy of renal masses: when and why. *Cancer imaging: the official publication of the International Cancer Imaging Society*.

[10] Pandey D., Garg P. K., Jakhetiya A. (2015). Surgical experience of primary salivary gland tumors of lung: a case series. *International Journal of Surgery*.

[11] Santamaría M., de Llano P. (2008). Adenoid cystic carcinoma metastatic to the kidney: a case report. *Acta Cytologica*.

[12] Kala S., Pantola C., Agarwal A. (2010). Metastatic adenoid cystic carcinoma of kidney masquerading as renal cell carcinoma. *Indian Journal of Pathology & Microbiology*.

[13] Bacalja J., Magazin M., Ulamec M., Rako D., Trnski D., Krušlin B. (2014). Adenoid cystic carcinoma of the lacrimal gland metastatic to the kidney: case report and review of the literature. *Scottish Medical Journal*.

[14] Qiu D. S., Xu L. Y., Hu X. Y. (2014). Imaging appearance of a singular metastatic adenoid cystic carcinoma of the right kidney: a case report and literature review. *Oncology Letters*.

[15] Chen L., Fang L., Zhang Y. (2016). Adenoid cystic carcinoma of maxillary sinus metastatic to the kidney: a case report and review of the literature. *International Journal of Clinical and Experimental Pathology*.

[16] Ladefoged C., Bisgaard C., Petri J. (2009). Solitary renal metastasis 23 years after extirpation of a bronchial adenoid cystic carcinoma. *Scandinavian Journal of Thoracic and Cardiovascular Surgery*.

[17] Fujii Y., Masuda M., Hirokawa M., Matsushita K., Hasegawa H. (1991). Bilateral renal metastases of lung adenoid cystic carcinoma. *Hinyokika Kiyo*.

[18] Goyal J., Sidana A., O'Malley M., Rodriguez R. (2011). Large renal metastasis from rare pulmonary neoplasm. *Urology*.

[19] Junejo N. N., Almusalam L., Alothman K. I., Al Hussain T. O. (2019). An unusual case report of pulmonary adenoid cystic carcinoma metastasis to the kidney. Case report and literature review. *Urology Case Reports*.

[20] Girelli L., Locati L., Galeone C. (2017). Lung metastasectomy in adenoid cystic cancer: is it worth it?. *Oral Oncology*.

[21] Dillon P. M., Chakraborty S., Moskaluk C. A., Joshi P. J., Thomas C. Y. (2016). Adenoid cystic carcinoma: a review of recent advances, molecular targets, and clinical trials. *Head & Neck*.

[22] Papaspyrou G., Hoch S., Rinaldo A. (2011). Chemotherapy and targeted therapy in adenoid cystic carcinoma of the head and neck: a review. *Head & Neck*.

[23] Ho A. L., Dunn L., Sherman E. J. (2016). A phase II study of axitinib (AG-013736) in patients with incurable adenoid cystic carcinoma. *Annals of Oncology*.

[24] Dillon P. M., Petroni G. R., Horton B. J. (2017). A phase II study of dovitinib in patients with recurrent or metastatic adenoid cystic carcinoma. *Clinical Cancer Research*.

[25] Tchekmedyian V., Sherman E. J., Dunn L. (2019). Phase II study of lenvatinib in patients with progressive, recurrent or metastatic adenoid cystic carcinoma. *Journal of Clinical Oncology*.

